# Improvement of cognitive function in wild-type and Alzheimer´s disease mouse models by the immunomodulatory properties of menthol inhalation or by depletion of T regulatory cells

**DOI:** 10.3389/fimmu.2023.1130044

**Published:** 2023-04-27

**Authors:** Noelia Casares, María Alfaro, Mar Cuadrado-Tejedor, Aritz Lasarte-Cia, Flor Navarro, Isabel Vivas, María Espelosin, Paz Cartas-Cejudo, Joaquín Fernández-Irigoyen, Enrique Santamaría, Ana García-Osta, Juan José Lasarte

**Affiliations:** ^1^ Immunology and Immunotherapy Program, Center for Applied Medical Research (CIMA), University of Navarra, Instituto de Investigación Sanitaria de Navarra (IdiSNA), Pamplona, Spain; ^2^ Gene Therapy for Neurological Disease Program, Center for Applied Medical Research (CIMA), University of Navarra, Instituto de Investigación Sanitaria de Navarra (IdiSNA), Pamplona, Spain; ^3^ Department of Pathology, Anatomy and Physiology, School of Medicine, University of Navarra, Pamplona, Spain; ^4^ Department of Radiology, Clínica Universidad de Navarra, University of Navarra, Instituto de Investigación Sanitaria de Navarra (IdiSNA), Pamplona, Spain; ^5^ Clinical Neuroproteomics Unit, Navarrabiomed, Hospital Universitario de Navarra (HUN), Universidad Pública de Navarra (UPNA), Instituto de Investigación Sanitaria de Navarra (IdiSNA), Pamplona, Spain

**Keywords:** immunomodulation, menthol, methimazole, olfactory system, Treg cells, central nervous system, cognitive capacity, Alzheimer´s disease

## Abstract

A complex network of interactions exists between the olfactory, immune and central nervous systems. In this work we intend to investigate this connection through the use of an immunostimulatory odorant like menthol, analyzing its impact on the immune system and the cognitive capacity in healthy and Alzheimer’s Disease Mouse Models. We first found that repeated short exposures to menthol odor enhanced the immune response against ovalbumin immunization. Menthol inhalation also improved the cognitive capacity of immunocompetent mice but not in immunodeficient NSG mice, which exhibited very poor fear-conditioning. This improvement was associated with a downregulation of IL-1β and IL-6 mRNA in the brain´s prefrontal cortex, and it was impaired by anosmia induction with methimazole. Exposure to menthol for 6 months (1 week per month) prevented the cognitive impairment observed in the APP/PS1 mouse model of Alzheimer. Besides, this improvement was also observed by the depletion or inhibition of T regulatory cells. Treg depletion also improved the cognitive capacity of the APP^NL-G-F/NL-G-F^ Alzheimer´s mouse model. In all cases, the improvement in learning capacity was associated with a downregulation of IL-1β mRNA. Blockade of the IL-1 receptor with anakinra resulted in a significant increase in cognitive capacity in healthy mice as well as in the APP/PS1 model of Alzheimer´s disease. These data suggest an association between the immunomodulatory capacity of smells and their impact on the cognitive functions of the animals, highlighting the potential of odors and immune modulators as therapeutic agents for CNS-related diseases.

## Graphical Abstract

Olfaction is becoming a central player in the pathophysiology of several brain disorders. Actually, smell may play a role in the prediction of the future brain diseases including Parkinson or Alzheimer. Also, the role of the immune system in these illnesses is a becoming issue to be considered as well. In our work we tried to shed light on the many intricate interactions between olfaction, immune system and nervous system. We have found that the stimulation of the immune system through exposure to certain fragrances, such as menthol, can have a beneficial effect on cognition. On the contrary, anosmia induced by methimazole treatment has a deleterious impact in cognitive capacity. We have demonstrated that menthol inhalation or Treg depletion improved cognitive skills in healthy mice and more importantly, in Alzheimer´s disease murine models. We have seen that downregulation of IL-1β expression in the brain is a common feature induced by menthol inhalation or Treg inhibition. Notably, IL-1 receptor inhibition by Anakinra administration improved cognitive capacity both in healthy and in an Alzheimer´s disease murine model. The link between impaired olfaction and immune system in neurologic diseases remains to be understood but it opens the door for its potential use in therapies for CNS-related diseases. (Created in Biorender.com).

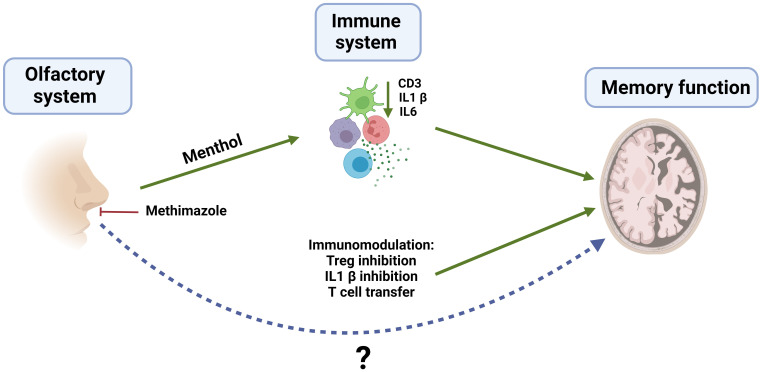

## 1 Introduction

There is evidence of the existence of a complex connection between the olfactory system, the central nervous system (CNS) and our cognitive behavior. In fact, various groups showed associations between the loss of olfactory function and CNS-related diseases including depression, schizophrenia, Alzheimer’s (AD) or Parkinson’s diseases. In some of these pathologies, olfactory dysfunctions precede the disease symptoms and are considered as a predictive factor (1–4).

Perception of smells may influence the physiological activity of the brain thus modulating brain functions including memory and/or emotions. The stimulation of olfactory receptors by the activation of guanine nucleotide binding proteins (GPCR), initiates synaptic signals that are transmitted to the brain by the olfactory bulb. Interestingly, this olfactory pathway presents direct connections to brain regions involved in memory and emotion such as the entorhinal cortex, hippocampus or amygdala, among others (reviewed in (5)). However, it is unknown if there is any other facilitator or intermediary element in this complex relationship between the olfactory system and the CNS. Apart from these more direct relationships between the olfactory system and the brain, the olfactory pathway may also affect the immune system, which is also somehow connected with the CNS. It was described that olfactory bulbectomy, commonly used as an animal model of depression (6), results in important immune changes (7). It remains unclear how the immune function underlies part of the brain abnormalities observed when the olfactory pathway is disrupted or which are the immune mediators involved. The truth is that there is increasing evidence indicating that the immune system plays a role in learning and memory, neural plasticity, brain functioning and behavioral processes (8–14).

In a previous study carried out in mice, we observed that certain odorants behaved as immunostimulatory or immunosuppressor agents. Interestingly, we found that carvone, an odor classified as immunosuppressive in C57BL/6J mice, also reduced their memory capacity (15). Exposure to menthol improved the immune response to antigen immunization. In this work we have confirmed the immunostimulatory properties of menthol and also discovered its beneficial effect on mice’s cognitive capacity. This surprising result encouraged us to study in more detail the possible interaction between the olfactory system, the immune system and the CNS.

We found that loss of olfactory capacity drastically reduced the immune response to immunization with a foreign antigen and worsened the memory capacity of mice. Because of their activity to modulate the immune system, we also studied the effect of the elimination of regulatory T cells (Treg) on mice’s cognitive capacity. Interestingly, we observed that repeated exposure to menthol or the inhibition or depletion of Treg reduced IL-1β mRNA levels in the brain and alleviated the age-related cognitive deterioration in AD mouse models. These improvements were also achieved by the administration of the IL-1 receptor inhibitor anakinra, pointing to this cytokine as a key player in the connection between the olfactory system, the immune system and the cognitive function. Despite the lack of a clear explanatory mechanism, our results constitute new evidence for the existence of a complex interaction among olfactory, immune and neurologic systems that may open new therapeutic opportunities for CNS-related diseases.

## 2 Materials and methods

### 2.1 Odorant stimulation system

Menthol (Aldrich Chemical Co., Milwaukee, WI.) was dissolved in distilled water (1:1000 w/v). A closed system prototype was designed to allow the vaporization of fragrance compounds (15). Inhalation of menthol was scheduled for different time periods (from 1 week to months depending on the experiments), with 8 cycles of 15 minutes of inhalation per day (1 cycle of exposure every 3 hours) as previously described (15). Schematics depicting the experimental procedures were created in Biorender.com.

### 2.2 Antigen presentation experiments

CD11c^+^ dendritic cells (DC) and CD8 T cells were obtained from the spleens of C57/BL6 and OT1 mice respectively, using magnetic separation columns according to the manufacturer’s specifications (Miltenyi Biotech, Germany). The purified CD11c^+^ or CD8^+^ T cells were used for antigen presentation experiments. Briefly, CD11c^+^ cells were incubated 2 hours at 37°C with 10 μg/ml of SIINFEKL peptide (Preprotech (UK). After washing, purified OTI CD8^+^ cells were added to the culture at a ratio 1:4 (CD11c: CD8). The co-culture was maintained for 24 hours to evaluate T cell proliferation (by [methyl-3H] thymidine incorporation) and IFNγ secretion (by ELISPOT) as previously described (15).

### 2.3 Mice and *in vivo* experiments

Six to eight-week-old BALB/c or C57BL/6 female mice (Envigo, Barcelona, Spain) were used to evaluate the impact of fragrance inhalation.

Male and female APP/PS1 mice were used to test the effect of menthol inhalation on memory. APP/PS1 (a recognized mouse model for AD (16, 17)) were bred and housed in the animal facility of the University of Navarra.

Male and female homozygous APP^NL-G-F^ mice (a new generation of AD mouse model (18)) were used to test the effect of Treg depletion in memory. The APP knock-in mouse (APP^NL-G-F/NL-G-F^) carries the humanized APP including three mutations associated with familial AD: the Swedish “NL”, the Iberian “F”, and the Arctic “G” mutation. The levels of pathogenic Aβ in this mouse model are elevated due to the combined effects of these three mutations that promote Aβ toxicity by increasing total Aβ production (Swedish mutation), rising the Aβ42/Aβ40 ratio (Iberian mutation), and promoting Aβ aggregation (Arctic mutation). These mutations lead to Aβ deposition and age-associated cognitive impairment (18). These APP^NL-G-F^ mice were provided by RIKEN Brain Science Institute (Japan). A colony was maintained and housed in the animal facility of the University of Navarra.

Male and female Foxp3-DTR-GFP transgenic mice (B6.129(Cg)-Foxp3tm3(DTR/GFP)Ayr/J) were obtained from Jackson Laboratory. DTR-eGFP expression is observed in fully functional Foxp3^+^CD4+ T cell populations allowing fluorescent detection of Foxp3^+^ Treg cells. This model allows for specific transient elimination of Foxp3^+^ Treg by treatment with diphtheria toxin. OTI transgenic mice (C57BL/6-Tg(TcraTcrb)1100Mjb/Crl) expressing a transgenic T cell receptor designed to recognize the H2K^b^-restricted cytotoxic T cell epitope from ovalbumin (residues 257-264) were purchased by Charles River. NSG mice were kindly provided by Dr. Melero (CIMA). All the experiments using male and female mice were balanced to avoid possible effects of sex. All these animals were inbred and housed in the animal facility of the University of Navarra.

All experiments were performed according to international animal care guidelines and with the approval of our local Ethics Committee for Animal Experimentation (Protocols 060c-19, 046-20, 087c-19, 097-20) and following the European Directive 2010/63/EU.

#### 2.3.1 Ovalbumin immunization experiments

C57BL/6 received an intravenous injection of chicken ovalbumin (OVA) protein (1 nmol/mouse) plus poly I:C (50 μg/mouse) and were exposed to vaporized menthol or water vapor as control for 7 days as previously described (15). Some groups were also treated with methimazole 75 mg/kg (i.p.) 6 hours before vaccination. Seven days after immunization, T cell proliferation (measured by methyl-3H-thymidine incorporation) and the measurement of IFN-γ producing T cells (carried out by ELISPOT using a kit from BD-Pharmingen (San Diego, CA) was conducted as previously described (15).

### 2.4 Contextual fear conditioning

Contextual fear conditioning test, a quick and reliable method to assess memory in rodents (19), was used to evaluate the mouse cognitive function as previously described (20). Briefly, the test takes 3 days. On day 1 (habituation), mice were placed in the training chamber for 3 min. Twenty-four hours later, day 2 (training phase), mice were placed in the training chamber for 2 min. Subsequently, mice received a footshock (0.3 mA 2 s) and returned to their home cage. Long-term memory was evaluated during the test phase at day 3 after training. Lack of movement was defined as freezing. Freezing scores were expressed as percentages. The procedure was carried out in a StartFear system (Panlab S.L., Barcelona, Spain). The analogical signal is transmitted to the FREEZING and STARTLE software. In T cell depletion experiments, C57BL/6 mice were treated intraperitoneally with 300μg/mouse of anti-CD25 antibody (BioXcell) four days before the fear conditioning test. Treg depletion was confirmed by flow cytometry. Specific depletion of Treg was induced in Foxp3-DTR mice by a single i.p. injection of 1 μg/mouse of diphtheria toxin 4 days prior behavioral studies. Depletion of Treg was confirmed by flow cytometry. Treg inhibition experiments were conducted by i.p injection of the FOXP3 inhibitor peptide CM1315 (produced by Wuxi App tech (China)) as previously described (21).

### 2.5 mRNA extraction and measurement of gene expression by iqPCR

Animals were perfused with PBS and prefrontal cortex and choroid plexus were dissected and frozen. RNA was purified and after the reverse transcription, cDNA amplification was performed on an Applied Biosystems CFX96 RT System using sense and antisense specific primers (**Supplementary Table 1**) as previously described (15). Relative expression of target genes was determined using the formula 2^ΔCt^, where ΔCt indicates the difference in the threshold cycle between the housekeeping gene (Cyclophilin A) and target genes.

### 2.6 Determination of Aβ levels

Brain Aβ_42_ levels (soluble and insoluble) were measured by using a sensitive sandwich ELISA kit (Invitrogen). Tissue (prefrontal cortex) was homogenized in a buffer containing 5 M guanidine HCl and 50 mM Tris-HCl, pH 8, protease inhibitors (Complete Protease Inhibitor Cocktail, Roche, Barcelona, Spain) and phosphatase inhibitors (0.1 mM Na3VO4, 1 mM NaF) and the assay was performed according to the manufacturer’s instructions.

### 2.7 Statistical analysis

Normality was assessed with Shapiro-Wilk W test. Statistical analyses were performed using parametric (Student´s t test and one-way ANOVA) and non-parametric (Mann-Whitney U and Kruskal-Wallis) tests. For all tests a *p* value <0.05 was considered statistically significant. Descriptive data for continuous variables are reported as means±SEM. GraphPad Prism for Windows was used for statistical analysis.

## 3 Results

### 3.1 Effect of the immunomodulatory properties of menthol in the cognitive capacity of mice

**Figure 1:**
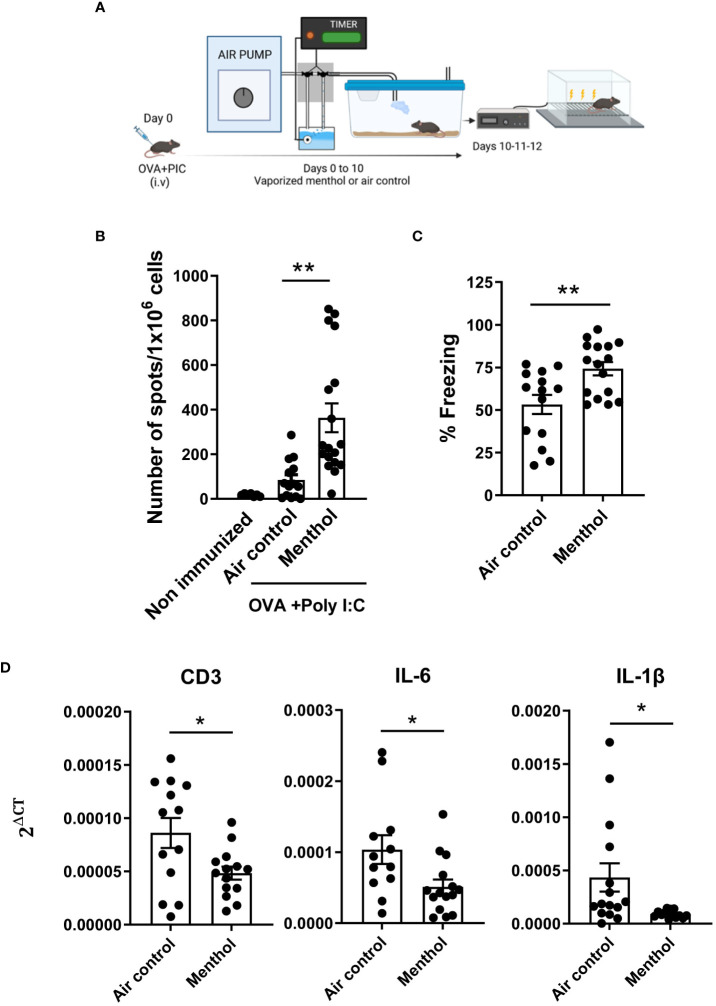
Effect of menthol inhalation in the immune response against ovalbumin immunization and on the cognitive capacity. **(A)** Schematic depicting the experimental procedure. Mice were immunized with OVA+ poly I:C and exposed to vaporized menthol or water vapor as control (Air control), for 7 days. **(B)** IFN-γ producing cells specific for SIINFEKL peptide were measured by ELISPOT. **(C)** Fear learning and memory were evaluated using a classic fear-conditioning assay. **(D)** mRNA for CD3, IL-1β or IL-6 was measured in mRNA samples obtained from prefrontal cortex of the indicated groups of mice. Data analyzed with student’s t-test. **p<0.01, *p<0.05.

To confirm the immunostimulatory properties of exposure to menthol (15), C57BL/6 mice (6 weeks old) were immunized with OVA mixed with poly I:C. Then, mice were housed in vaporization cages and exposed to cycles of 15 min of menthol or water vapor (air control) every 3 h during 7 days (8 cycles per day, 15 min/cycle, n=12-15 mice per group). Ten days after immunization, mice were evaluated in their cognitive capacity by using the fear conditioning test (**Figure 1A**). After this analysis, mice were sacrificed to evaluate the immune response against the OVA cytotoxic T cell epitope SIINFEKL measuring the number of IFN-γ-producing cells by ELISPOT (**Figure 1B**). Mice exposed to menthol had significantly higher numbers of IFN-γ producing cells specific for SIINFEKL peptide. Notably, mice exposed to menthol exhibited significantly more freezing than control mice, suggesting an improvement in their cognitive capacity (**Figure 1C**). Interestingly, mRNA expression levels in the prefrontal cortex indicated a significant reduction of CD3 as well as IL-6 and IL-1β in mice exposed to menthol compared to control mice (**Figure 1D**), two cytokines that have been associated with cognitive decline in humans (9, 22, 23).

### 3.2 Effect of methimazole-induced anosmia on memory capacity in mice

**Figure 2:**
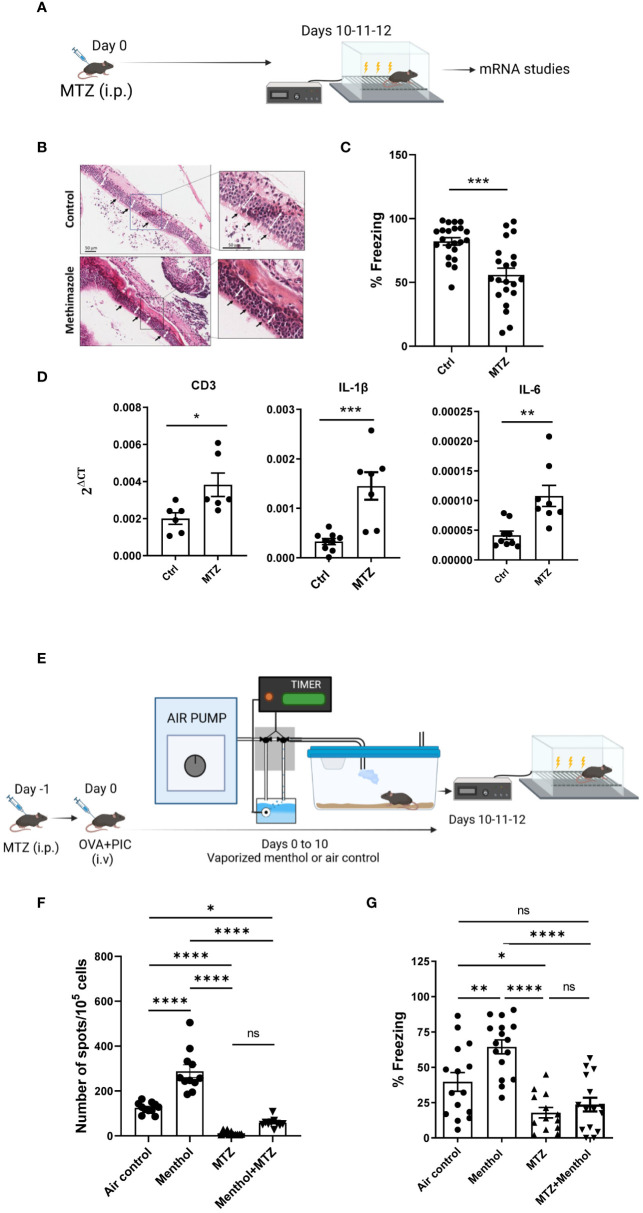
Effect of methimazole (MTZ) treatment in cognitive capacity and the immune system in C57BL/6 mice. **(A)** Schematic depicting the experimental procedure analyzed in **(B–D)** C57BL/6 female mice were treated with a single injection of MTZ i.p. and 10 days later, olfactory epithelia were isolated and stained by H&E **(B)**. Cilia atrophy (black arrows) and epithelial thickness (white arrows) in the olfactory epithelium from methimazole treated mice compared to the epithelium of control mice is indicated. **(C)** Fear memory evaluated in mice treated or not with MTZ. **(D)** mRNA for CD3, IL-1β or IL-6 was measured in mRNA samples obtained from prefrontal cortex of the indicated groups of mice. **(E)** Schematic depicting the experimental procedure analyzed in F and **(G)** Naïve or MTZ treated C57BL/6 mice were immunized with ovalbumin plus Poly I:C. and seven days after immunization IFN-γ producing cells specific for SIINFEKL peptide measured by ELISPOT **(F)**. **(G)** Fear conditioning assay for the indicated groups of mice. Data were analyzed with student’s t-test **(C, D)** and one-way ANOVA **(F, G)**. ****p<0.0001, ***p<0.005, **p<0.01, *p<0.05. ns, not significant.

In an attempt to go deeper into this complex interaction, we studied the effect of anosmia on the immunomodulatory/cognitive effects of menthol (**Figure 2A**). It was reported that methimazole (MTZ) administration produces extensive degenerative changes in olfactory epithelium and a severe deficit in odor detection in rats or mice (24–26). We treated the mice with MTZ or with saline (n=20-24 mice per group) and ten days later, the olfactory epithelium was isolated and analyzed by H&E staining. As previously described, cilia atrophy (black arrows) and epithelial thickening (white arrows) in the olfactory epithelium was observed (**Figure 2B**). When a fear conditioning test was carried out in mice 10 days after a single injection of MTZ, a reduction in the percentage of freezing values was observed in anosmic mice, suggesting impairment in their cognitive capacity (**Figure 2C**). This impairment in memory capacity was associated with an increase in CD3, IL-6 and IL-1β mRNA expression in the prefrontal cortex of the mice (measured in a subgroup of 6-8 randomly selected mice, **Figure 2D**).

We then studied the potential impact of MTZ on the immune system by measuring its effects in mice after immunization with OVA (n=12-15 mice per group, **Figure 2E**). Although this immunization induced high numbers of IFN-γ producing cells specific for the cytotoxic T cell epitope SIINFEKL, this effect was dramatically impaired when mice were treated with a single dose of MTZ after immunization with OVA. Moreover, exposure to menthol odor in MTZ treated mice did not restore the immunogenicity of OVA previously observed (**Figure 2F**). Importantly, immune inhibition induced by MTZ treatment was accompanied by an impaired cognitive capacity, which was not recovered by exposure to menthol (**Figure 2G**). Freezing values in control mice in the experiments plotted in **Figures 2C, G** are different. This is probably due to the differences in the protocol and the environment to which the animals were subjected in both settings (27, 28). Despite these differences in both control groups, it can be concluded in both experiments that MTZ treatment negatively affects cognitive ability measured in the fear conditioning experiment. These data may suggest that anosmia causes immunosuppression, and has a deleterious effect on memory capacity.

To discard potential unspecific immunosuppressive effects of MTZ, we tested its effect on T cells and DCs *in vitro* and *in vivo*. Splenocytes isolated from naïve mice were stimulated with anti-CD3 antibodies in the presence/absence of 50, 10 or 1 μM of MTZ and T cell proliferation was measured. No significant changes in proliferation were observed upon MTZ treatment, suggesting that MTZ does not have a direct detrimental effect on T cells (**Supplementary Figure 1A**). We then treated naïve mice with 75 mg/kg of MTZ or saline and, one week later, splenocytes were stimulated *in vitro* with anti-CD3 antibodies to measure T cell proliferation. No significant differences were observed in the proliferative capacity of T cells (**Supplementary Figure 1B**). Similarly, we could not detect any inhibitory effect of MTZ on DC *in vitro* or *in vivo*. DCs isolated from naïve C57BL/6 mice and treated *in vitro* with 100 or 500 μM of MTZ and then pulsed with SIINFEKL peptide, stimulated OT1 T cell proliferation and IFN-γ production similarly to untreated control DC (**Supplementary Figure 1C**). Likewise, DCs purified from MTZ-treated mice and then pulsed with SIINFEKL peptide showed the same antigen presentation capacity as DC from untreated mice (**Supplementary Figure 1D**). DCs purified from MTZ or saline-treated mice responded similarly to LPS stimulation (not shown). These data indicate that MTZ has no direct impact on immune cells.

### 3.3 Effect of the immune system on memory capacity in mice.

**Figure 3:**
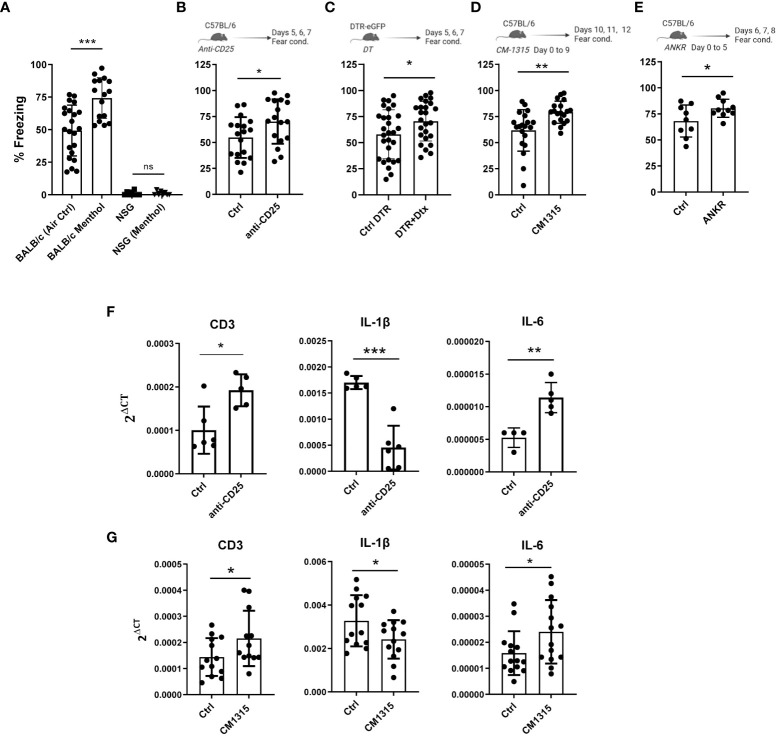
Role of the immune system and its modulation in mice cognitive capacity measured by contextual fear conditioning test. Fear memory was evaluated in wt BALB/c mice as well as in the immunodeficient NSG mice exposed to menthol or vehicle (air control) **(A)**, in C57BL/6 mice treated with anti-CD25 antibodies **(B)**, in Foxp3-DTR mice treated with diphtheria toxin (Dtx) to deplete Treg cells **(C)**, in C57BL/6 mice treated with the Treg inhibitory peptide CM1315 **(D)** and in C57BL/6 mice treated with the IL-1 receptor antagonist anakinra (ANKR) **(E)**. **(F, G)** mRNA for CD3, IL-1β or IL-6 was measured in mRNA samples obtained from brain prefrontal cortex of mice treated with anti-CD25 antibodies **(F)** or with the Treg inhibitor CM1315 **(G)**. Data were analyzed with one-way ANOVA **(A)** and with student’s t-test **(B–G)**. ***p<0.005, **p<0.01, *p<0.05.

Once the link between the olfactory and the immune system was established, we aimed to study the effect of the immune system on memory capacity. First, we evaluated the fear memory of the highly immunodeficient NSG mice (NOD scid gamma mouse strain, with a mixed background between BALB/c and C57BL/6) lacking T cells, B cells and natural killer cells. BALB/c and C57BL/6 showed a similar % of freezing in the fear conditioning test (**Supplementary Figure 2A**). While BALB/c mice had normal freezing values that were improved by menthol exposure, NSG mice had a dramatic memory impairment that was not affected by menthol vaporization (n=10-15 mice per group, **Figure 3A**). These data suggest that immune cells, either those absent (T, B or NK cells) or those defective (monocytes or macrophages) in NSG mice could have a role in learning function. Indeed, there is accumulating evidences on the implication of cytokines produced by immune cells in the behavioral response to stimuli, cognition and learning but also in the context of neurodegenerative diseases (reviewed in (13)). To evaluate the role of immune T cell activation, we carried out *in vivo* experiments of depletion or inhibition of T regulatory cells, a key specific T cell subpopulation maintaining immune tolerance by controlling the activation of T cells (29). In fact, it was suggested that the depletion of Treg cells might mitigate the neuroinflammatory response in murine models of AD and reverse the cognitive decline observed in these animals (30). Thus, C57BL/6 mice were treated with anti-CD25 antibodies four days before the fear conditioning experiment (n=16 mice per group). Interestingly, Treg depletion significantly improved mice’s cognitive capacity (**Figure 3B**). Similar results were found when using *Foxp3DTR* knock-in mice treated with diphtheria toxin to deplete Foxp3^+^ Treg cells (n=25 mice per group, **Figure 3C**) or when mice were treated with the Treg inhibitory peptide CM1315 (31) (n=20 mice per group, 100 μgr/mouse per day, **Figure 3D**). Diphtheria toxin administration in C57BL/6 wt mice does not affect cognitive capacity (**Supplementary Figure 2B**). Baruch et al. showed that transient Treg depletion affects the brain’s choroid plexus, a selective gateway for immune cell trafficking to the CNS (30). Thus, we studied the levels of expression of CD3, IL-6, and IL-1β mRNA expression in the choroid plexus (measured in a subgroup of 4-6 randomly selected mice). Interestingly, we found that CD25 cell depletion resulted in higher CD3 mRNA levels, suggesting an increase in T cell infiltration into the brain. Moreover, we found a reduction in IL-1β and an increase in IL-6 mRNA levels (**Figure 3F**). Similar results were found in mice treated with the Treg inhibitory peptide CM1315 (**Figure 3G**). These data suggest that IL-1β can have a detrimental effect on a fear-motivated learning task, results in agreement with previous observations in different murine models (32). Notably, we observed that intraperitoneal administration of the IL-1β inhibitor anakinra for 6 consecutive days (50 mg/kg per day) significantly improved the freezing time in our system (n=10 mice per group, **Figure 3E**), suggesting a beneficial role of IL-1β blockade in learning capacity.

### 3.4 Effect of Treg depletion on the cognitive function in APP^NL-G-F^ mouse model of AD

**Figure 4:**
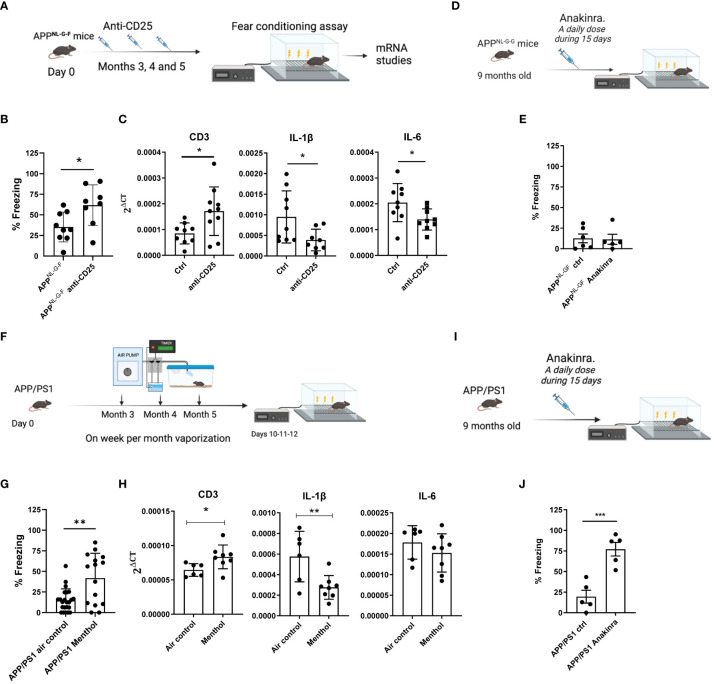
Effect of Treg depletion or menthol inhalation in the cognitive capacity in Alzheimer´s disease murine models. **(A)** Schematic depicting the experimental procedure analyzed in **(B)** and **(C)**. APP^NL-G-F^ mice were treated with anti-CD25 antibodies at months 3, 4 and 5 after birth and 1 week after the last injection, memory capacity (fear conditioning) was evaluated in both groups **(B)**. **(C)** mRNA levels for CDR3, IL-1β or IL-6 in the brain prefrontal cortex were evaluated by RT-PCR. **(D)** Schematic depicting the experimental procedure analyzed in **(E)**. APP^NL-G-F^ mice were treated with anakinra (ANKR) or with saline for 15 consecutive days before the fear conditioning assay **(E)**. **(F)** Schematic depicting the experimental procedure analyzed in **(G)** and **(H)**. APP/PS1 tg mice were exposed to menthol or water vapor for 6 weeks in total, 1 week per month, with 8 cycles of 15 minutes of inhalation per day. **(G)** A fear conditioning experiment was performed at the end of the experiment to evaluate the effect of menthol on memory capacity. **(H)** mRNA for CD3, IL-1β or IL-6 was measured in mRNA samples obtained from brain prefrontal cortex of the indicated groups of mice. **(I)** Schematic depicting the experimental procedure analyzed in **(J)**. APP/PS1 mice were treated with anakinra or with saline for 15 consecutive days before the analysis of their memory capacity **(J)**. Data were analyzed with student’s t-test. **p<0.01, *p<0.05.

Prompted by the positive effects of Treg depletion in memory tasks, we tested the effect of Treg depletion in the behavior of 5-month-old APP^NL-G-F^ mice, already described to suffer an age-dependent memory impairment (33) (**Supplementary Figure 2C**). APP^NL-G-F^ mice were treated with saline or with anti-CD25 antibodies at months 3, 4 and 5 (one administration of 300 μg/mouse per month, n= 9-10 mice per group). Treg depletion was analyzed in the peripheral blood 6 days after each antibody administration. One week after the last anti-CD25 treatment at month 5, mice were evaluated in the freezing conditioning paradigm (**Figure 4A**). Interestingly, it was found that mice treated with the anti-CD25 antibody had a significantly higher percentage of freezing, suggesting an improvement in their cognitive capacity (**Figure 4B**). This improvement was associated with a significant increase in CD3 and a significant decrease of IL-1β and IL-6 mRNA in the prefrontal cortex (**Figure 4C**). This decrease of IL-1β and IL-6 mRNA was also observed in the choroid plexus (**Supplementary Figure 3**). We then explored the effect of anti-CD25 on Aβ pathology in APP^NL-G-F^ mice. Total Aβ42 levels (soluble and insoluble) in the prefrontal cortex of these animals were determined by a sandwich ELISA. As shown in **Supplementary Figure 4A**, no differences were observed in Aβ42 levels in APP^NL-G-F^ mice treated with anti-CD25 compared with vehicle-treated mice.

We analyzed the effect of anakinra in a new cohort of APP^NL-G-F^, (n=5-6 mice per group) but this time we used 9-month-old mice (having a very significant cognitive decline). Mice were treated with 15 consecutive doses of anakinra (one administration of 50 mg/kg per day) (**Figure 4D**) and evaluated later in the fear conditioning assay. Unfortunately, we could not see a beneficial effect of the IL-1β inhibition in this experimental setting (**Figure 4E**).

### 3.5 Effect of immune modulation by menthol on the cognitive function in APP/PS1 mice

Once observed the effects of the immune cells on the memory of both young mice and the APP^NL-G-F^ mice and considering the effect of menthol on the immune system and cognitive capacity of healthy naïve mice, we aimed to study the effect of menthol inhalation in a different AD mouse model. In this case, we used the APP/PS1 mice that develop beta-amyloid deposits in the brain at 4 months of age, having a slower progression of the disease than the APP^NL-G-F^ mice but having also a cognitive decline at this age (**Supplementary Figure 2D**). In this 6-month-long experiment, APP/PS1 mice were exposed to menthol or water vapor for 6 weeks in total, 1 week per month, with 8 cycles of 15 minutes of inhalation per day (n=15-16 mice per group). A fear conditioning experiment was performed at the end of the experiment to compare the effect of menthol versus water vapor inhalation (**Figure 4F**). As previously described, APP/PS1 mice treated with water vapor (air control) showed a poor performance in fear-conditioning. However, 1-week exposure to menthol per month, during these 6 months was able to significantly prevent the cognitive impairment observed in the APP/PS1 mice treated with water vapor reaching freezing values similar to wild-type control mice (**Figure 4G**). Interestingly, the memory improvement was again associated with a significant reduction of IL-1β mRNA in the brain cortex of the animals exposed to menthol (measured in a subgroup of 6-8 randomly selected mice, **Figure 4H**). We also analyzed the effect of anakinra in a new cohort of 9-month-old APP/PS1 mice. Mice were treated with 15 consecutive doses of anakinra (one administration per day) (n=5 mice per group, **Figure 4I**) and evaluated later in the fear conditioning assay. Surprisingly, the blockade of IL-1β with anakinra in the APP/PS1 model achieved a very significant improvement in cognitive capacity in this experimental setting (**Figure 4F**).

In order to determine whether memory recovery observed in menthol APP/PS1 treated mice was associated with an amelioration of amyloid pathology, total levels of Aβ42 were determined in the prefrontal cortex of these animals. As shown in **Supplementary Figure 4B**, exposure to menthol did not reduce Aβ42 levels, indicating that the mechanism of improvement in cognitive ability is independent of this parameter.

## 4 Discussion

Complex interactions among brain cells with immune functions (like microglia and astrocytes), peripheral immune cells (T cells, NK cells and macrophages), neurons, and neural precursor cells participate in the physiological homeostasis of the brain. Activation of the immune system by infections or chronic stressful conditions, with the release of cytokines and other mediators, may have direct effects on memory, neural plasticity and neurogenesis (34, 35) having also an important role in CNS diseases. Initially, inflammation is associated with sickness behavior and infiltration of peripheral immune cells into the CNS with pathological results. However, an accumulating body of research has pointed to neuroimmune interactions as primarily beneficial, in that they promote homeostasis of the nervous system (36–42). Accordingly, any intrinsic or extrinsic factors that might alter the brain immune environment may also have an impact on the CNS behavior (9).

In this complex network of interactions, we investigated the potential role that the olfactory system may have in the immune system and on the CNS. Several reports have addressed the immunomodulatory and/or neurological effects of odorants (15, 43–49). In a previous exploratory experiment using an array of different volatile compounds we found that menthol was an immunostimulatory odor in C57BL/6 mice (15). We have now confirmed this finding and more interestingly, we observed that exposure to menthol improves the cognitive capacity of healthy young mice.

We also found that the impairment of olfaction induced by MTZ treatment had a clear impact on the cognitive capacity measured by fear conditioning test. It is remarkable that the improvement of cognitive capacity by menthol was correlated with a reduction in the expression of IL-6 and IL-1β as well as CD3 mRNA in the brain cortex, whereas MTZ treatment had the opposite effect.

There is some controversy on the role of these cytokines on learning and memory, with evidences for both negative and positive effects (reviewed in (9, 50). Impairment of cognitive tasks in mice deficient in IL-1R1 (8), IL-6 (10) or TNFR2 (12), clearly support the concept of the need of cytokine signaling to maintain homeostatic behavior (13). Notably, IL-1β or IL-6 appear upregulated in AD and other neurodegenerative disorders (23, 51–53) and are correlated with cognitive decline (54–56). Our results suggest that the neurological effect of menthol could be mediated by changes in the expression of these cytokines, especially IL-1β. Several studies point at IL-1β as a key player in CNS pathological conditions but also in regulating physiological neuroplasticity in the generation of memory (reviewed in (57)). However, contradictory effects have been reported for IL-1β with important activities in neurogenesis but also in neuronal apoptosis, affecting the synaptic structure and the neuronal excitability (reviewed in (58)). Despite all this controversy, there is an increasing interest for exploring IL-1R1 as a therapeutic target in CNS diseases. There are several IL-1R1-antagonizing biological therapeutics (biologics such as anakinra or anti-IL-1β antibodies) showing correlations with substantial benefit in cerebral autoinflammatory diseases (59) or cognitive improvement in AD murine models (60). In our experiments, we found a beneficial effect of anakinra treatment on the percentage of freezing of healthy mice suggesting a negative role for IL-1β in learning task. More importantly, although we were not able to see an improvement in the cognitive capacity in 9 months-old APP^NL-G-F^ mice by anakinra administration during 15 consecutive days, this treatment achieved a very significant improvement in cognitive capacity in the APP/PS1 model. Similar results were obtained in a previous publication by Kitazawa et al. (60), with the administration of anti–IL-1R blocking mAb to 9-month-old 3xTg-AD mice every 8–9 days for 6 months. The lack of efficacy of anakinra in the APP^NL-G-F^ could be related to the aggressiveness of this model, that it has been shown to develop a more accelerated AD, with a more important amyloid pathology than the APP/PS1 model. Probably a longer dosing schedule of the IL-1β inhibitor could have a more measurable effect on cognitive activity in this more aggressive model of AD. Even though more in-depth studies are required to confirm this finding, it could be hypothesized that IL-1β inhibitors or treatments that regulate its expression, can have beneficial roles in CNS diseases such as AD. There are other cytokines, apart from IL-1β or IL-6 that might play a fundamental role in cognition and behavior (13). Among them is IFNγ, that has been identified as an important factor regulating neuronal connectivity and social behavior (61), playing a relevant role in cognitive dysfunction and memory related processes in healthy mice and in AD murine models (62–64) as well as in patients with Alzheimer (65). We found by RT-PCR an increase of the expression of mRNA for IL-1β and IL-6 as well as for IFN-γ, (**Supplementary Figure 5**) pointing to these cytokines, as targets for modulation in cognitive decline. Likewise, it is reasonable to consider that some immunomodulatory agents affecting these cytokines may play a beneficial role in this disease.

The connection between the immune system and cognitive capacity was also evidenced in the highly immunosuppressed NSG mouse strain that showed a clear impairment in fear-conditioning learning task. These results suggest a direct or indirect role of lymphocytes or NK cells in the learning process. Notably, depletion of Treg caused a clear benefit in cognitive capacity of healthy young mice that was associated with an increase in CD3 and a decrease in IL-1β expression, suggesting again that this cytokine could be behind the beneficial effect of the lack of Treg and the change in inflammatory microenvironment in the brain.

Treg cells are known to cause both beneficial and detrimental influences in central nervous system pathologies including AD among others (66). In AD in particular, a sustained neuroinflammation and a high expression of inflammatory mediators is known to be associated with increased neurodegeneration (67) and as such, Treg cells might play an important role in the progression of the disease. However, their precise contribution is complex and remains unclear. There are works showing an aggravation of spatial learning deficits after Treg depletion in AD animal models (68), while others describe a reversal of cognitive decline (30). In the same line, adoptive transfer of Treg cells or the administration of IL-2 to expand Treg improve synaptic plasticity and restore cognitive function in AD animal models (69, 70) whereas other studies suggest a deleterious role for Treg (30, 71, 72). We have found a beneficial role of Treg depletion in the memory capacity in healthy young mice and, more importantly, in a mouse model of AD. This improvement was associated with a reduction of IL-1β and IL-6 in the pre-frontal brain cortex as well as in the choroid plexus. The expression levels of these cytokines in other brain regions not directly associated with memory formation has not been explored in this study, but it would be interesting to assess the impact of Treg inhibition or even the menthol inhalation in other CNS activities. In the same way that other studies have failed to find a correlation between a decrease in amyloid pathology and memory improvement in AD models (73–76), memory improvement observed with both immunomodulatory therapeutic approaches in AD-mice was not associated with an amelioration of amyloid burden. This result might indicate that the mechanism of improvement in cognitive ability would be independent of this parameter. In fact, amyloid plaques in humans do not necessarily correlate with memory function (77).

Although further studies are needed to clarify the exact contribution and phenotype of Treg in AD, it is becoming evident that disruption of the immune system functioning leads to impairments in cognition and neurogenesis. Our results could be in agreement with the hypothesis that a certain level of immunostimulation might have a beneficial role in brain fitness (13, 40, 78–80), thereby providing a therapeutic opportunity for CNS-related diseases.

Our results in the APP/PS1 model, where we found a beneficial effect of repeated exposure to menthol during the long process taking the development of cognitive impairment, are in line with this hypothesis. It is important to highlight at this point the correlation described between the processes of anosmia with a faster rate of cognitive decline that precedes the appearance of the first symptoms in Alzheimer’s disease (1–3). Whether loss of olfaction predicts or is behind the AD-related neurodegenerative changes warrants further investigation. If it were the latter, it would be interesting to evaluate the impact of strategies to improve the olfactory ability of the patients (81). In accordance with this idea, it has been recently shown that intensive olfactory training can improve olfactory function and that this improvement is associated with changes in the structure of olfactory processing areas of the brain (82–84).

In summary, our data suggest that those immunomodulatory strategies able to modulate the expression of cytokines, in particular IL-1β, might have an impact on cognitive functions. These data highlight the potential of the immunomodulatory properties of odors and other immunomodulators as therapeutic agents for CNS-related diseases (**Graphical abstract**). Our findings in the APP/PS1 murine model require confirmation with further studies in greater depth, but they open the door to the development of therapies based on the stimulation and training of the olfactory system to prevent or alleviate the effects of this devastating CNS disease.

## Data availability statement

The raw data supporting the conclusions of this article will be made available by the authors, without undue reservation.

## Ethics statement

The animal study was reviewed and approved by Ethics Committee for Animal Experimentation, Universidad de Navarra.

## Author contributions

This study was designed, directed and coordinated by JL, NC, AG-O and MC-T. MC-T and AG-O, provided conceptual and technical guidance for neurological aspects of the study and were implicated in the interpretation of data. MA, AL-C, ME, IV, FN, PC-C, JF-I, ES, JL and NC provided conceptual and technical guidance and were implicated in the interpretation of data. MA and NC conducted most of the experiments of this study. The manuscript was initially written by JL and reviewed and edited by all authors. All authors contributed to the article and approved the submitted version.
