# Supplementary material for: Improvement of cognitive function in wild-type and Alzheimer´s disease mouse models by the immunomodulatory properties of menthol inhalation or by depletion of T regulatory cells
Source: Front Immunol. 2023 Apr 27;14:1130044. doi: 10.3389/fimmu.2023.1130044 (PMC10175945; doi:10.3389/fimmu.2023.1130044)
Supplement: Supplementary file 1 [file DataSheet_1.pdf]

Supplementary information.

Immunomodulation by Menthol inhalation or T regulatory cell depletion improve the cognitive function in wild type and Alzheimer´s disease mouse models

Noelia Casares[1]*, María Alfaro[1]*, Mar Cuadrado-Tejedor[2,3], Aritz Lasarte-Cia[1], Flor Navarro[1], Isabel Vivas[4], María Espelosin[2], Paz Cartas-Cejudo[5], Joaquín Fernández-Irigoyen[5], Enrique Santamaría[5], Ana García-Osta[2†], Juan José Lasarte[1†.]

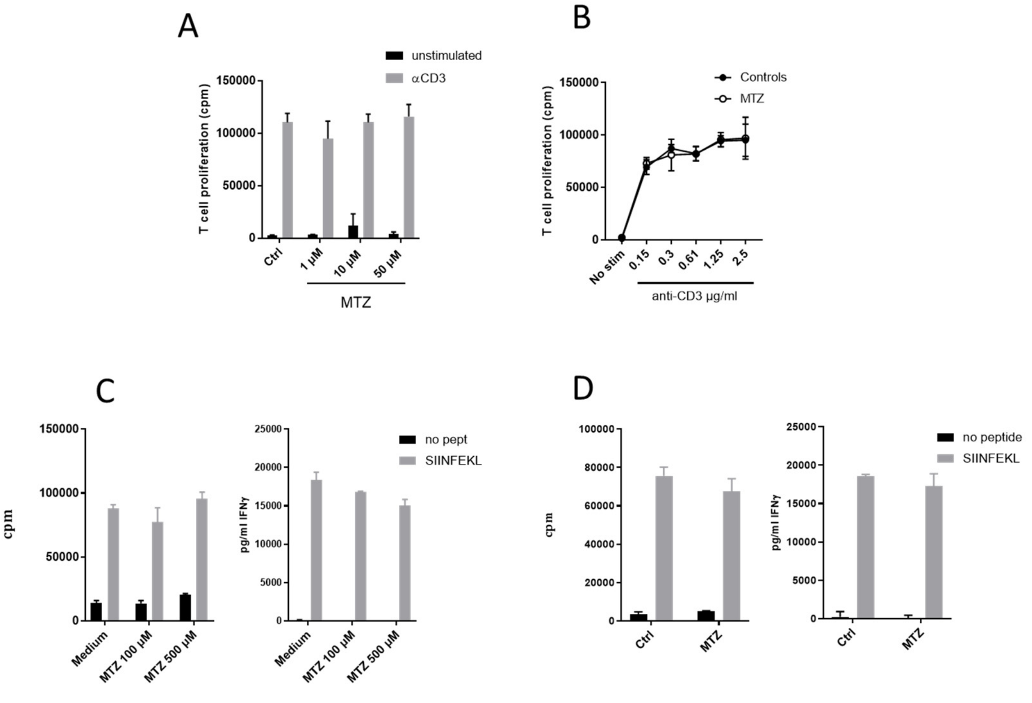

Supplementary Fig 1. Effects of MTZ on the immune system. (A) Spleens from C57 BL/6 mice were cultured *in vitro* in the presence of different concentrations of MTZ and stimulated with aCD3ε antibodies (2.5 μg/ml, BD Biosciences). Two days later, proliferation was measured by thymidine incorporation. (B) C57 BL/6 mice were injected with 75 mg/kg of MTZ i.p. One week later, mice were sacrificed and splenocytes were stimulated *in vitro* with different concentrations of anti-CD3 antibodies to measure T cell proliferation by thymidine incorporation. (C) DC cells (CD11c+) isolated from spleen were pulsed with SIINFEKL peptide for 2 hours at 37ºC. Then, CD8+ OT-1 T cells were added to the culture (ratio 1:4, DCs: CD8) in the presence/absence of the indicated concentrations of MTZ. Twenty four hours later T cell proliferation and IFN-γ production were measured. (D) DC cells (CD11c+) were purified from the spleen of mice treated with MTZ (75 mg/kg) one week before. DC were pulsed with SIINFEKL peptide 2 hours at 37ºC. Later, CD8+ OT-1 T cells were added to the culture (ratio 1:4, DCs: CD8). T cell proliferation and IFN-γ production were analysed.

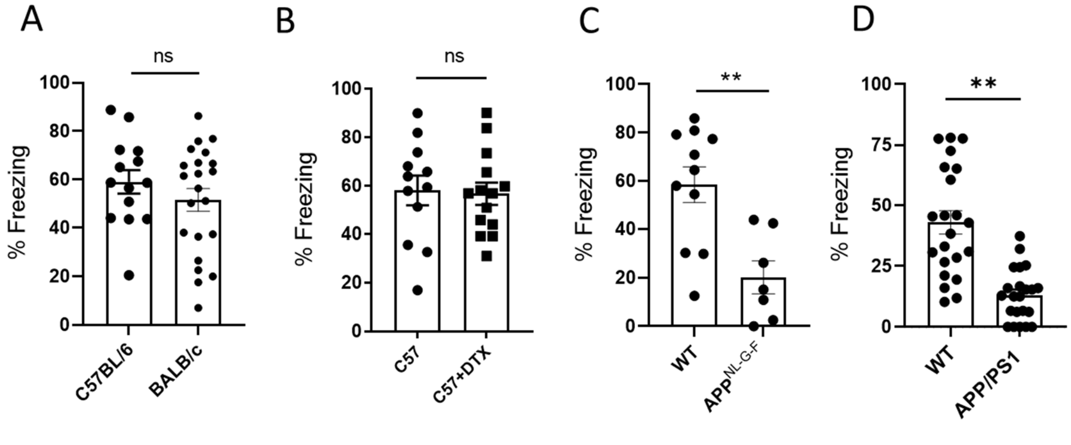

Supplementary Fig 2. Results of fear conditioning in the different animal models. (A) Comparison of the % of freezing in wt C57BL/6 and BALB/c mice, (B) in C57BL/6 mcie treated with saline or with diphtheria toxin, (C) in wt or in APP$^{NL\text{-}G\text{-}F}$ transgenic mice and (D) in wt or in APP/PS1 transgenic mice.

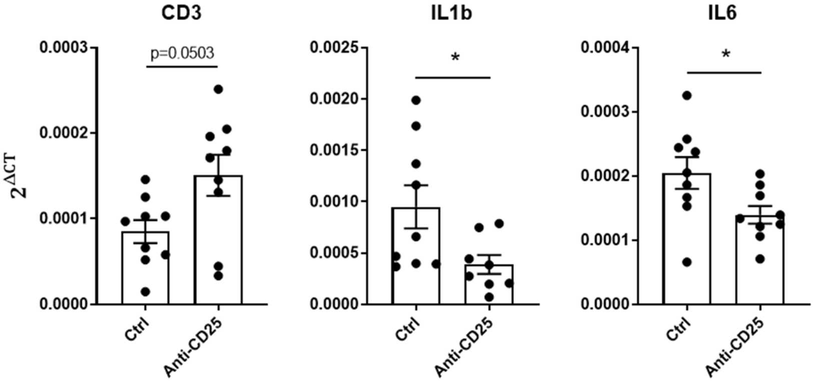

Supplementary Fig 3. Levels of mRNA for CD3, IL-1β or IL-6 measured in mRNA samples obtained from the choroid plexus of APP$^{NL\text{-}G\text{-}F}$ **mice treated with anti-DC25 antibodies.** Data were analysed with student's t-test. *p<0.05.

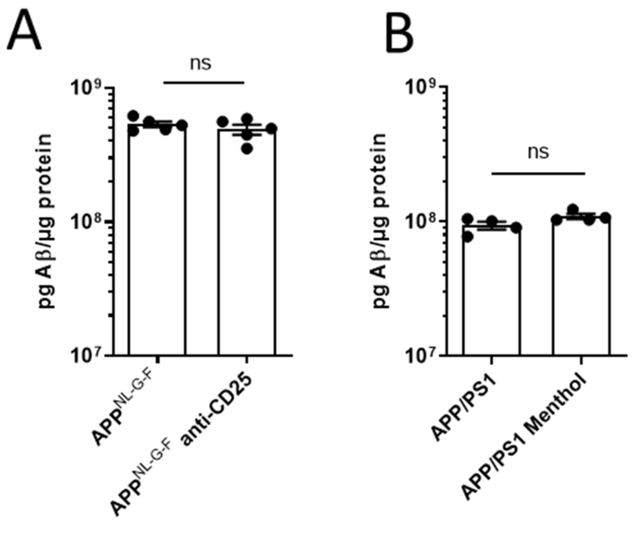

Supplementary Figure 4. Brain Aβ42 levels in the prefrontal cortex of APP^{NL-G-F} transgenic mice treated with anti CD25 antibodies or with saline 8A) and in APP/PS1 transgenic mice exposed to menthol or to air control (B) as described in Figures 4A and 4F respectively. The tissue was homogenized in specific buffers and Aβ42 levels were quantified by ELISA. Data were analysed with student's t-test. *p<0.05.

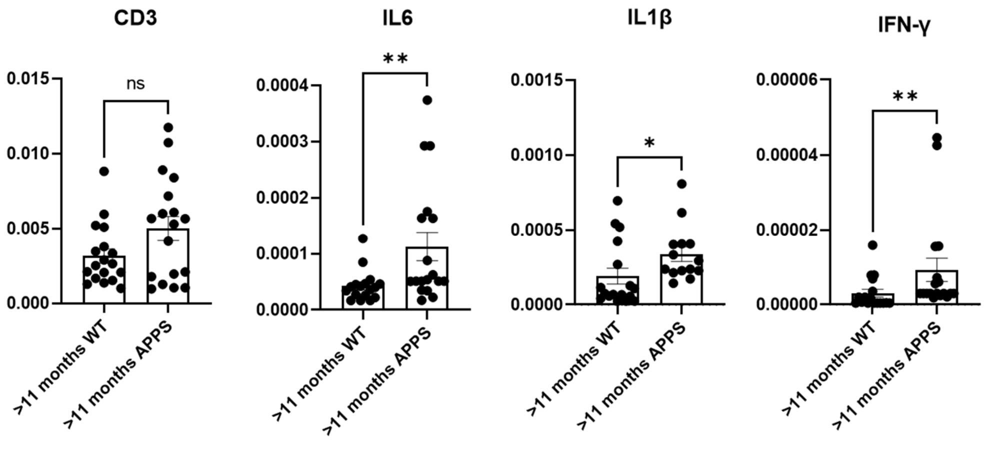

Supplementary Figure 5. Quantitation of cytokine mRNA expression in the prefrontal cortex tissue from APP/PS1 mice and control littermates. Relative expression of mRNA for CD3, IL6, IL1b and IFN-γ genes in APP/PS1 and WT mouse models at >11 months of age, measured by real time PCR. The housekeeping gene used to normalize the mRNA expression levels was cyclophilin A. Data were analysed with student's t-test. **p<0.01, *p<0.05.

Supplementary Table 1

Sequence of primers used to measure gene expression by iQPCR

mIL6-s	ACAAAGCCAGAGTCCTTCAG
mIL6-a	TGGATGGTCTTGGTCCTTAG
mIL1beta-s	GCCACCTTTTGACAGTGATG
mIL1beta-a	TAATGGGAACGTCACACACC
mCD3-s	TGACTCCCAAATCAATGTG
mCD3-a	GCAGGTGAAGCTTGTCTG
mIFNγ1-s	TCAAGTGGCATAGATGTGGAAGAA
mIFNγ1-a	TGGCTCTGCAGGATTTTCATG
mCyclophilin-s	AGCATACAGGTCCTGGCATC
mCyclophilin-a	TTCACCTTGCCAAAGACCAC
